# Crystal structure of potassium sodium hepta­hydrogen hexa­molybdocobaltate(III) octa­hydrate: an extra-protonated *B*-series Anderson-type heteropolyoxidometalate

**DOI:** 10.1107/S2056989015014784

**Published:** 2015-08-12

**Authors:** Ki-Min Park, Hea-Chung Joo, Uk Lee

**Affiliations:** a Research Institute of Natural Science, Gyeongsang National University, 501 Jinju-daero, Jinju, 660-701, Republic of Korea; b Department of Chemistry, Pukyong National University, 599-1 Daeyeon 3-dong, Nam-gu, Busan 608-737, Republic of Korea

**Keywords:** crystal structure, novel protonated *B* series Anderson-type polyanion, hexa­molybdocobaltate(III), noncentrosymmetric strong hydrogen bond

## Abstract

The title compound, KNa[Co^III^(OH)_7_{Mo_6_O_17_}]·8H_2_O, was obtained by the ion-exchange technique from K_3_[Co(μ_3_-OH)_6_Mo_6_O_18_]·7H_2_O. Six μ_3_-O atoms and one O atom of the bridging μ_2_-O atom are protonated. This novel polyanion protonated by an extra H atom is an unexpected polyanion species among the *B*-series Anderson-type polyoxidometalates (POMs), [*X^n^* ^+^(μ_3_-OH)_6_Mo_6_O_18_]^(6–*n*)–^] (*X* = heteroatom). The extra H atom (seventh H atom) in the polyanion does not lie on a crystallographic centre of symmetry, but is located at the mid-point between two μ_2_-O atoms of adjacent polyanions, and forms a very short hydrogen bond [2.430 (5) Å]. The present structure is considered as particularly significant in understanding noncentrosymmetric strong hydrogen bonding.

## Abstract 2

A novel hexa­molybdate(VI) polyanion protonated by an extra H atom is an unexpected polyanion species among the *B*-series Anderson-type polyoxometalates (POMs). The extra (seventh) H atom does not lie on a crystallographic centre of symmetry, but is located at the mid-point between two μ_2_-O atoms of adjacent polyanions, and forms a very short hydrogen bond.

## Chemical context   

The six H atoms attached to the μ_3_-O atoms of the central [*X*O_6_] (*X* = heteroatom) octa­hedron in *B*-series Anderson-type heteropolyoxidomolybdates (Anderson, 1937 ▸; Tsigdinos, 1978 ▸), [*X^n^* ^+^(μ_3_-OH)_6_Mo_6_O_18_]^(6–*n*)–^] [*X* ^n+^ = Ni^2+^ (Lee *et al.*, 2002 ▸), Cu^2+^ (Ito *et al.*, 1989 ▸), Al^3+^ (Lee *et al.*, 1991 ▸), Cr^3+^ (Perloff, 1970 ▸), Co^3+^ (Nolan *et al.*, 1998 ▸; Lee *et al.*, 2001 ▸), Rh^3+^ (Ozawa *et al.*, 1991 ▸)], are non-acidic (*i.e.* nondissociative). For the past four decades, the existence of a protonated species with more than seven H^+^ ions was not expected for this class of compounds; the supposed highest number of seven was shown by K_2_[H_7_Cr^III^Mo_6_O_24_]·8H_2_O (Joo *et al.*, 2015*a* ▸). A free-acid type compound, H_3_[H_6_AlMo_6_O_24_]·10H_2_O (Liu *et al.*, 2006 ▸), was reported but the positions of protonated O atoms by the excess three H^+^ ions were not defined. The current study was carried out to confirm the presence of a highly protonated species that exists at very low pH.

Considering the geometry of the inter­polyanion hydrogen bonds by an extra H atom (seventh H atom), observed *via* electron-density maps around the protonated μ_2_-O*B* atoms and bond valence sums (BVSs; Brown & Altermatt, 1985 ▸; Brese & O’Keeffe, 1991 ▸) of the protonated μ_2_-O*B* atoms in the polyanion, we can determine that the positions of the extra H atoms follow a pseudosymmetric model in the polyanion. Sometimes a short hydrogen bond (O⋯O 〈 2.60 Å), in which the H atom lies on a crystallographic centre of symmetry, occurs in this class of structure (Lee *et al.*, 2010 ▸; Joo *et al.*, 2015*b* ▸). The focus of this report is to clarify the position of the extra H atom of the polyanion in the title compound.

## Structural commentary   

**Figure 1:**
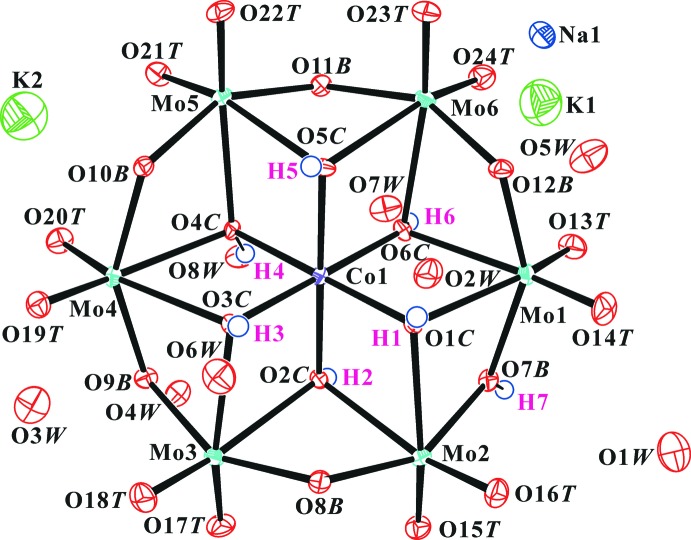
The polyanion structure and the cations as well as the lattice water molecules in the title compound. Displacement ellipsoids are drawn at the 50% probability level for non-H atoms. H atoms are drawn as small spheres of arbitrary radius.

Fig. 1 ▸ shows the the components of the crystal structure of the title compound. The O atoms of the heteropolyanion have been designated as O*T* (terminal Mo=O atom), O*B* (bridging μ_2_-O*B* atom; Mo—O—Mo), and O*C* (centred μ_3_-O atom; Mo_2_—O*C*—Co). The protonated O atoms in the polyanion were confirmed by the BVSs, the charge balance, the bond-length elongation and the inter­polyanion hydrogen bonds (Fig. 3 ▸ and Table 1 ▸).

**Figure 2:**
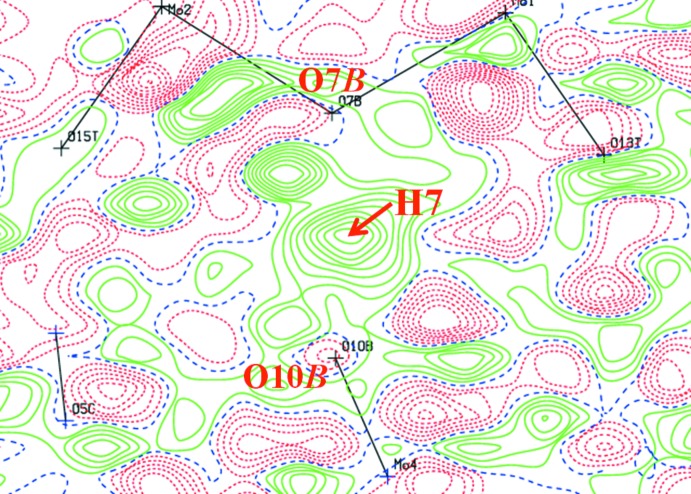
Difference Fourier map between atoms O7*B* and O10*B*, where H atoms were absent.

Consider the symmetry relation of O7*B* and O10*B* atoms, the electron density of the H atom between atoms O7*B* and O10*B* in the difference Fourier map (Fig. 2 ▸) and the very short O7*B*⋯O10*B* distance of 2.430 (5) Å. Also consider the bond elongations by protonation of Mo1/2—O7*B* and Mo4/5—O10*B*, and the bond angles of Mo—O*B*—Mo. These data suggest that O7*B* or O10*B* in the polyanion should be protonated.

**Figure 3:**
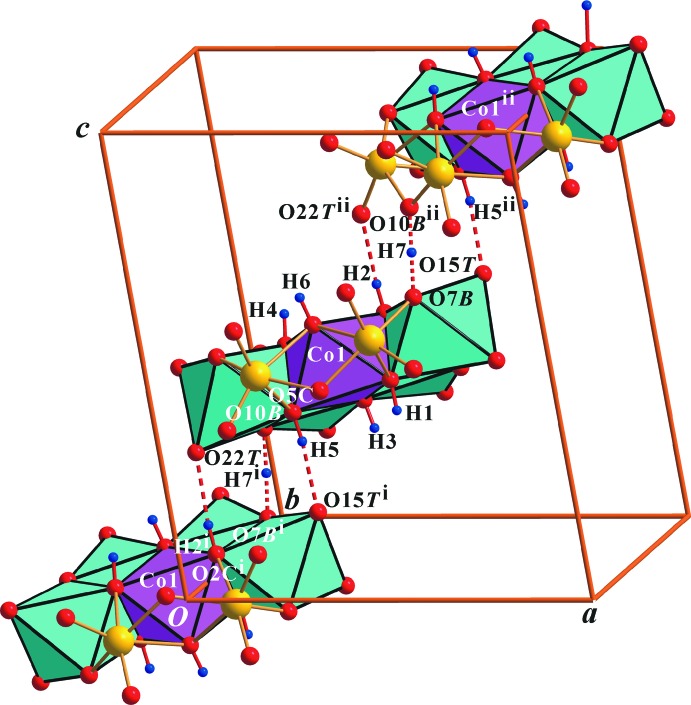
Polyhedral view with unit cell of the heteropolyanion in the title compound, with the O⋯O contacts of the inter­polyanion hydrogen bonds shown as dashed lines. [Symmetry codes: (i) *x* − 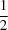, −*y* + 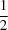, *z* − 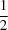; (ii) *x* + 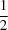, −*y* + 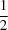, *z* + 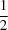.]

Confirmation of the protonated O atom was strongly supported by the BVS analysis. The calculated BVSs for expected protonation atoms O7*B* and O10*B* are 1.63 and 1.61 valence units (v.u.), respectively, if the valence of the O—H bond is not included. Since the BVS value around the O atom should be 2.0 v.u., the missing valences of O7*B* and O10*B* are 0.37 and 0.39 v.u., respectively, which corresponds to the valence of the O—H bonds. The BVS values for the unprotonated O8*B*, O9*B*, O11*B* and O12*B* atoms are 1.98, 1.94, 1.95 and 1.95 v.u., respectively. The reasonable BVSs of short and long O—H bond lengths can be obtained from the graphical correlation valences (Brown, 2002 ▸). This showed that atom H7 in the polyanion has a distance of 1.21 Å with 0.41 v.u. As a result, the valence sums around O7*B* and O10*B* are 2.04 and 2.01 v.u., respectively. Therefore, these valence unit values satisfy the protonation conditions of O7*B* and O10*B* atoms in the polyanion. As a result, these data suggest that H7 is located on the midpoint between O7*B* and O10*B* ^ii^ atoms (the symmetry code corresponds to that in Fig. 3 ▸). However, the H7 atom contributes to the short hydrogen bonds, and does not lie on a crystallographic centre of symmetry; also, the electron density is not symmetric in the polyanion (Fig. 2 ▸), although we expect H7 atom to lie in the middle of the bond, which corresponds to a pseudosymmetric short hydrogen bond. This means that an extra H atom is co-shared by an adjacent polyanion; for example, μ_2_-O7*B*⋯H7⋯μ_2_-O10*B* ^ii^ (Fig. 3 ▸).

The BVSs for the K1, K2, and Na1 ions are 0.50, 0.55, and 1.26 v.u, respectively, in the title compound (Na⋯O 〈 2.50 Å and K⋯O 〈 3.00 Å). BVS calculations for K1 and K2 reveal a considerable under-saturation in terms of valence units, which we ascribe to the disordered character of the K^+^ position. All the BVSs agree well with the charge-balance requirements. The K^+^ ions are coordinated by four and three O atoms as [K1(O*W*)(O*B*)(O*T*)_2_]^+^ and [K2(O*W*)_2_(O*T*)_2_]^+^. The Na^+^ ion is coordinated by six O atoms as [Na1(O*W*)_4_(O*T*)_2_]^+^.

## Supra­molecular features   

The polyanions are linked together into chains along [101] *via* hydrogen bonds: two normal inter-polyanion μ_3_-O (O*C*)⋯μ_1_-O (O*T*) and one very short μ_2_-O7*B*–H7⋯μ_2_-O10*B* bond (Fig. 3 ▸ and Table 1 ▸). Note that water mol­ecules O6*W*, O7*W* and O8*W* do not show any inter­action with the metal atoms and are bonded to other O atoms only by hydrogen bonds. The other H atoms of the polyanion, (H1, H3, H4 and H6) form hydrogen bonds with water mol­ecules (Table 1 ▸).

## Synthesis and crystallization   

Title compound was obtained from the ion-exchanged solution (*ca* pH 1.4) of K_3_[H_6_CoMo_6_O_24_]·7H_2_O (Lee *et al.*, 2001 ▸) by Amberlite IR120. The resulting solution was concentrated in a hot water bath. After 1 d, stable blue crystals were obtained at room temperature. The Na^+^ ion in the title compound is considered to have been a contaminant from the ion-exchange resin.

## Refinement   

The crystal data, the data collection and the structure refinement details are summarized in Table 2 ▸. All H atoms in the polyanion and all H atoms in the water mol­ecules were located from difference Fourier maps. All H atoms of the polyanion were refined with a distance restraint of O—H = 0.85 (3) Å, except O7*B*–H7, and were included in the refinement with *U*_iso_(H) = 1.5*U*_eq_(O). The bond lengths of O7*B*—H7 and O10*B*—H7^i^ (the symmetry code corresponds to that in Fig. 3 ▸) were constrained by using the SADI (σ = 0.03) command; they were set to be equal with an effective standard uncertainty to locate the shared H atom on the pseudocentre between atoms O7*B* and O10*B*. The H atoms of all the water mol­ecules (O*W*) were refined with distances and angles restraints of O—H = 0.85 (3) Å and H*A*⋯H*B* = 1.35 (3) Å, and were included in the refinement with *U*_iso_(H) = 1.5*U*_eq_(O). Reasonable displacement ellipsoids of K1 and K2 were obtained with half-occupancy.

## Supplementary Material

Crystal structure: contains datablock(s) New_Global_Publ_Block, I. DOI: 10.1107/S2056989015014784/br2252sup1.cif

Structure factors: contains datablock(s) I. DOI: 10.1107/S2056989015014784/br2252Isup2.hkl

CCDC reference: 1417467

Additional supporting information:  crystallographic information; 3D view; checkCIF report

## Figures and Tables

**Table 1:** Hydrogen-bond geometry (, )

*D*H*A*	*D*H	H*A*	*D* *A*	*D*H*A*
O1*C*H1O2*W*	0.85(3)	1.81(3)	2.639(6)	167(7)
O2*C*H2O22*T* ^i^	0.81(3)	1.98(3)	2.787(6)	170(7)
O3*C*H3O6*W*	0.84(3)	1.99(4)	2.775(6)	154(6)
O4*C*H4O8*W*	0.84(3)	1.81(3)	2.627(6)	165(7)
O5*C*H5O15*T* ^ii^	0.83(3)	1.99(3)	2.822(6)	171(7)
O6*C*H6O7*W*	0.82(3)	1.96(3)	2.761(6)	165(7)
O7*B*H7O10*B* ^i^	1.21(2)	1.22(2)	2.430(5)	175(6)
O1*W*H1*B*O14*T*	0.86(3)	1.89(4)	2.731(7)	163(8)
O1*W*H1*A*O16*T*	0.85(3)	2.18(5)	2.878(7)	140(6)
O2*W*H2*A*O8*W* ^iii^	0.85(3)	1.91(3)	2.757(6)	176(7)
O2*W*H2*B*O15*T* ^ii^	0.83(3)	2.13(4)	2.841(6)	145(6)
O3*W*H3*B*O19*T*	0.83(3)	2.01(3)	2.792(7)	156(6)
O3*W*H3*A*O1*W* ^ii^	0.83(3)	2.02(4)	2.784(7)	154(8)
O4*W*H4*A*O23*T* ^i^	0.84(3)	1.97(3)	2.800(6)	167(7)
O4*W*H4*B*O9*B*	0.84(3)	1.91(3)	2.734(6)	167(7)
O6*W*H6*B*O3*W*	0.84(3)	1.88(3)	2.709(7)	169(7)
O6*W*H6*A*O11*B* ^iii^	0.83(3)	2.31(6)	2.921(6)	131(6)
O7*W*H7*A*O8*B* ^iv^	0.81(3)	2.42(6)	2.937(6)	122(6)
O7*W*H7*B*O6*W* ^iv^	0.82(3)	2.00(3)	2.811(7)	166(8)
O8*W*H8*B*O4*W*	0.83(3)	1.88(3)	2.697(7)	168(7)
O8*W*H8*A*O7*W*	0.82(3)	2.01(4)	2.761(8)	151(7)

Symmetry codes: (i) 
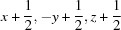
; (ii) 
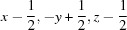
; (iii) 
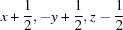
; (iv) 
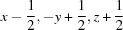
.

**Table 2:** Experimental details

Crystal data	
Chemical formula	KNa[CoMo_6_O_17_(OH)_7_]8H_2_O
*M*_r_	1231.84
Crystal system, space group	Monoclinic, *P*2_1_/*n*
Temperature (K)	173
*a*, *b*, *c* ()	10.9758(5), 20.7702(9), 12.7906(6)
()	99.666(1)
*V* (^3^)	2874.5(2)
*Z*	4
Radiation type	Mo *K*
(mm^1^)	3.37
Crystal size (mm)	0.20 0.10 0.05

Data collection	
Diffractometer	Bruker SMART APEXII CCD diffractometer
Absorption correction	Multi-scan (*SADABS*; Bruker, 2009 ▸)
*T*_min_, *T*_max_	0.669, 0.838
No. of measured, independent and observed [*I* > 2(*I*)] reflections	17925, 6701, 4416
*R*_int_	0.043
(sin /)_max_ (^1^)	0.666

Refinement	
*R*[*F* ^2^ > 2(*F* ^2^)], *wR*(*F* ^2^), *S*	0.041, 0.081, 1.06
No. of reflections	6701
No. of parameters	449
No. of restraints	31
H-atom treatment	Only H-atom coordinates refined
_max_, _min_ (e ^3^)	1.02, 1.06

Computer programs: *APEX2* (Bruker, 2009 ▸), *SAINT* (Bruker, 2009 ▸), *SHELXS97* (Sheldrick, 2008 ▸), *SHELXL2014* (Sheldrick, 2015 ▸), *ORTEP-3 for Windows* (Farrugia, 2012 ▸), *PLATON* (Spek, 2009 ▸) and *DIAMOND* (Brandenburg, 1998 ▸).

## supplementary crystallographic information

### Crystal data

**Table:**

KNa[CoMo_6_O_17_(OH)_7_]·8H_2_O	*F*(000) = 2352
*M**_r_* = 1231.84	*D*_x_ = 2.846 Mg m^−^^3^
Monoclinic, *P*2_1_/*n*	Mo *K*α radiation, λ = 0.71073 Å
*a* = 10.9758 (5) Å	Cell parameters from 5031 reflections
*b* = 20.7702 (9) Å	θ = 2.5–28.2°
*c* = 12.7906 (6) Å	µ = 3.37 mm^−^^1^
β = 99.666 (1)°	*T* = 173 K
*V* = 2874.5 (2) Å^3^	Block, blue
*Z* = 4	0.20 × 0.10 × 0.05 mm

### Data collection

**Table:**

Bruker SMART APEXII CCD diffractometer	6701 independent reflections
Radiation source: rotating anode	4416 reflections with *I* > 2σ(*I*)
Detector resolution: 10.0 pixels mm^-1^	*R*_int_ = 0.043
φ and ω scans	θ_max_ = 28.3°, θ_min_ = 1.9°
Absorption correction: multi-scan (*SADABS*; Bruker, 2009)	*h* = −14→14
*T*_min_ = 0.669, *T*_max_ = 0.838	*k* = −27→10
17925 measured reflections	*l* = −16→16

### Refinement

**Table:**

Refinement on *F*^2^	Secondary atom site location: difference Fourier map
Least-squares matrix: full	Hydrogen site location: difference Fourier map
*R*[*F*^2^ > 2σ(*F*^2^)] = 0.041	Only H-atom coordinates refined
*wR*(*F*^2^) = 0.081	*w* = 1/[σ^2^(*F*_o_^2^) + (0.0192*P*)^2^ + 12.1333*P*] where *P* = (*F*_o_^2^ + 2*F*_c_^2^)/3
*S* = 1.06	(Δ/σ)_max_ = 0.001
6701 reflections	Δρ_max_ = 1.02 e Å^−^^3^
449 parameters	Δρ_min_ = −1.06 e Å^−^^3^
31 restraints	Extinction correction: *SHELXL2014* (Sheldrick, 2015), Fc^*^=kFc[1+0.001xFc^2^λ^3^/sin(2θ)]^-1/4^
Primary atom site location: structure-invariant direct methods	Extinction coefficient: 0.00011 (2)

### Special details

**Table:**

Geometry. All esds (except the esd in the dihedral angle between two l.s. planes) are estimated using the full covariance matrix. The cell esds are taken into account individually in the estimation of esds in distances, angles and torsion angles; correlations between esds in cell parameters are only used when they are defined by crystal symmetry. An approximate (isotropic) treatment of cell esds is used for estimating esds involving l.s. planes.

### Fractional atomic coordinates and isotropic or equivalent isotropic displacement parameters (Å^2^)

**Table:**

	*x*	*y*	*z*	*U*_iso_*/*U*_eq_	Occ. (<1)
Mo1	0.53782 (5)	0.10915 (3)	0.53624 (4)	0.01162 (13)	
Mo2	0.71702 (5)	0.24276 (3)	0.53614 (4)	0.01042 (12)	
Mo3	0.58803 (5)	0.38359 (3)	0.47083 (4)	0.01109 (12)	
Mo4	0.28516 (5)	0.39351 (3)	0.39378 (4)	0.01036 (12)	
Mo5	0.10877 (5)	0.25896 (3)	0.38934 (4)	0.01082 (12)	
Mo6	0.23869 (5)	0.11899 (3)	0.45827 (4)	0.01178 (13)	
Co1	0.41250 (8)	0.25144 (4)	0.46414 (7)	0.00890 (16)	
K1	0.3893 (4)	0.0424 (2)	0.2202 (3)	0.0427 (10)	0.5
K2	−0.0640 (5)	0.4290 (3)	0.5040 (4)	0.0659 (14)	0.5
Na1	0.1547 (3)	0.01011 (12)	0.7104 (2)	0.0225 (6)	
O1C	0.5469 (4)	0.1996 (2)	0.4361 (3)	0.0099 (9)	
H1	0.552 (6)	0.192 (3)	0.372 (3)	0.015*	
O2C	0.5375 (4)	0.2937 (2)	0.5617 (3)	0.0093 (9)	
H2	0.530 (6)	0.292 (3)	0.624 (3)	0.014*	
O3C	0.4390 (4)	0.3205 (2)	0.3718 (3)	0.0086 (9)	
H3	0.455 (6)	0.310 (3)	0.312 (3)	0.013*	
O4C	0.2764 (4)	0.3033 (2)	0.4915 (3)	0.0093 (9)	
H4	0.285 (6)	0.307 (3)	0.557 (2)	0.014*	
O5C	0.2891 (4)	0.2085 (2)	0.3653 (3)	0.0108 (9)	
H5	0.299 (6)	0.210 (3)	0.302 (3)	0.016*	
O6C	0.3851 (4)	0.1826 (2)	0.5565 (3)	0.0108 (9)	
H6	0.365 (6)	0.181 (3)	0.615 (3)	0.016*	
O7B	0.6477 (4)	0.1741 (2)	0.6169 (3)	0.0111 (9)	
H7	0.664 (6)	0.171 (3)	0.7129 (13)	0.017*	
O8B	0.6943 (4)	0.3130 (2)	0.4423 (3)	0.0125 (9)	
O9B	0.4270 (4)	0.4140 (2)	0.4939 (3)	0.0120 (9)	
O10B	0.1788 (4)	0.3281 (2)	0.3095 (3)	0.0109 (9)	
O11B	0.1319 (4)	0.1898 (2)	0.4860 (3)	0.0117 (9)	
O12B	0.3973 (4)	0.0895 (2)	0.4323 (3)	0.0127 (9)	
O13T	0.5124 (4)	0.0676 (2)	0.6458 (4)	0.0198 (11)	
O14T	0.6444 (4)	0.0669 (2)	0.4824 (4)	0.0210 (11)	
O15T	0.8019 (4)	0.2762 (2)	0.6482 (3)	0.0162 (10)	
O16T	0.8163 (4)	0.1981 (2)	0.4768 (3)	0.0176 (10)	
O17T	0.6726 (4)	0.4151 (2)	0.5825 (3)	0.0194 (11)	
O18T	0.6115 (4)	0.4313 (2)	0.3682 (3)	0.0191 (11)	
O19T	0.3029 (4)	0.4388 (2)	0.2856 (3)	0.0152 (10)	
O20T	0.1757 (4)	0.4314 (2)	0.4505 (3)	0.0179 (10)	
O21T	0.0045 (4)	0.3034 (2)	0.4433 (3)	0.0158 (10)	
O22T	0.0288 (4)	0.2243 (2)	0.2763 (3)	0.0152 (10)	
O23T	0.1523 (4)	0.0867 (2)	0.3471 (3)	0.0185 (10)	
O24T	0.2177 (4)	0.0719 (2)	0.5621 (4)	0.0195 (11)	
O1W	0.8736 (5)	0.0678 (3)	0.4260 (4)	0.0275 (12)	
H1A	0.889 (6)	0.1078 (16)	0.427 (6)	0.041*	
H1B	0.796 (3)	0.067 (3)	0.430 (6)	0.041*	
O2W	0.5288 (4)	0.1640 (2)	0.2360 (4)	0.0182 (10)	
H2A	0.601 (3)	0.169 (3)	0.223 (5)	0.027*	
H2B	0.484 (5)	0.187 (3)	0.193 (5)	0.027*	
O3W	0.4675 (4)	0.4437 (3)	0.1413 (4)	0.0241 (11)	
H3A	0.450 (6)	0.452 (4)	0.077 (2)	0.036*	
H3B	0.403 (4)	0.445 (4)	0.168 (5)	0.036*	
O4W	0.4278 (4)	0.4240 (2)	0.7072 (3)	0.0184 (10)	
H4A	0.497 (4)	0.416 (4)	0.744 (4)	0.028*	
H4B	0.436 (6)	0.426 (4)	0.643 (2)	0.028*	
O5W	0.3599 (5)	0.0204 (3)	0.7925 (4)	0.0372 (14)	
H5A	0.391 (8)	0.048 (3)	0.757 (5)	0.056*	
H5B	0.385 (8)	0.029 (4)	0.857 (2)	0.056*	
O6W	0.5384 (5)	0.3204 (2)	0.1861 (4)	0.0217 (11)	
H6A	0.526 (7)	0.303 (3)	0.127 (3)	0.033*	
H6B	0.526 (7)	0.3600 (14)	0.176 (5)	0.033*	
O7W	0.2948 (4)	0.1969 (3)	0.7441 (4)	0.0247 (12)	
H7A	0.322 (6)	0.188 (4)	0.805 (3)	0.037*	
H7B	0.222 (3)	0.185 (4)	0.730 (5)	0.037*	
O8W	0.2654 (4)	0.3256 (2)	0.6919 (3)	0.0193 (11)	
H8A	0.284 (6)	0.293 (2)	0.728 (5)	0.029*	
H8B	0.319 (5)	0.354 (2)	0.705 (5)	0.029*	

### Atomic displacement parameters (Å^2^)

**Table:**

	*U*^11^	*U*^22^	*U*^33^	*U*^12^	*U*^13^	*U*^23^
Mo1	0.0123 (3)	0.0096 (3)	0.0125 (3)	0.0008 (2)	0.0008 (2)	0.0011 (2)
Mo2	0.0084 (2)	0.0118 (3)	0.0107 (3)	−0.0002 (2)	0.0006 (2)	0.0000 (2)
Mo3	0.0110 (2)	0.0106 (3)	0.0112 (3)	−0.0017 (2)	0.0005 (2)	0.0006 (2)
Mo4	0.0111 (2)	0.0097 (3)	0.0103 (3)	0.0016 (2)	0.0018 (2)	0.0003 (2)
Mo5	0.0089 (2)	0.0133 (3)	0.0098 (3)	−0.0002 (2)	0.0002 (2)	−0.0002 (2)
Mo6	0.0117 (3)	0.0112 (3)	0.0119 (3)	−0.0029 (2)	0.0004 (2)	0.0016 (2)
Co1	0.0090 (3)	0.0093 (4)	0.0084 (3)	0.0008 (3)	0.0013 (3)	0.0002 (3)
K1	0.045 (2)	0.038 (2)	0.044 (2)	0.0036 (19)	0.0044 (18)	−0.0032 (18)
K2	0.063 (3)	0.068 (4)	0.070 (3)	0.007 (3)	0.021 (3)	−0.006 (3)
Na1	0.0272 (14)	0.0186 (14)	0.0212 (13)	−0.0012 (13)	0.0029 (11)	−0.0004 (13)
O1C	0.011 (2)	0.009 (2)	0.010 (2)	−0.0001 (18)	0.0010 (18)	−0.0016 (18)
O2C	0.011 (2)	0.008 (2)	0.008 (2)	−0.0004 (17)	−0.0007 (18)	0.0012 (18)
O3C	0.013 (2)	0.009 (2)	0.004 (2)	−0.0002 (18)	0.0027 (17)	−0.0003 (17)
O4C	0.010 (2)	0.009 (2)	0.009 (2)	0.0023 (17)	0.0029 (18)	−0.0010 (18)
O5C	0.010 (2)	0.016 (2)	0.006 (2)	−0.0056 (18)	0.0015 (18)	−0.0016 (18)
O6C	0.014 (2)	0.010 (2)	0.008 (2)	−0.0010 (18)	0.0041 (18)	0.0010 (18)
O7B	0.013 (2)	0.013 (2)	0.006 (2)	0.0014 (18)	−0.0027 (17)	−0.0005 (17)
O8B	0.011 (2)	0.014 (2)	0.014 (2)	0.0002 (18)	0.0057 (18)	0.0032 (18)
O9B	0.012 (2)	0.013 (2)	0.010 (2)	−0.0009 (18)	0.0009 (17)	−0.0053 (18)
O10B	0.012 (2)	0.011 (2)	0.009 (2)	0.0002 (18)	−0.0007 (17)	0.0010 (17)
O11B	0.011 (2)	0.013 (2)	0.013 (2)	0.0011 (18)	0.0051 (18)	0.0001 (18)
O12B	0.011 (2)	0.014 (2)	0.012 (2)	−0.0031 (18)	−0.0013 (18)	−0.0013 (18)
O13T	0.021 (3)	0.019 (3)	0.018 (2)	−0.004 (2)	−0.001 (2)	0.006 (2)
O14T	0.020 (2)	0.020 (3)	0.023 (3)	0.007 (2)	0.006 (2)	−0.007 (2)
O15T	0.015 (2)	0.019 (3)	0.013 (2)	−0.002 (2)	−0.0006 (19)	0.0007 (19)
O16T	0.017 (2)	0.015 (3)	0.021 (2)	0.005 (2)	0.006 (2)	0.001 (2)
O17T	0.015 (2)	0.023 (3)	0.018 (2)	−0.002 (2)	−0.0047 (19)	−0.003 (2)
O18T	0.024 (3)	0.018 (3)	0.017 (2)	0.000 (2)	0.006 (2)	0.007 (2)
O19T	0.016 (2)	0.015 (2)	0.014 (2)	−0.0007 (18)	0.003 (2)	0.0028 (19)
O20T	0.016 (2)	0.020 (3)	0.018 (2)	0.002 (2)	0.0050 (19)	−0.002 (2)
O21T	0.014 (2)	0.016 (2)	0.019 (2)	−0.0010 (19)	0.0082 (19)	−0.003 (2)
O22T	0.013 (2)	0.020 (3)	0.012 (2)	−0.0023 (19)	−0.0017 (18)	0.0009 (19)
O23T	0.016 (2)	0.017 (3)	0.021 (2)	−0.005 (2)	−0.001 (2)	−0.004 (2)
O24T	0.018 (2)	0.021 (3)	0.019 (2)	−0.002 (2)	0.003 (2)	0.003 (2)
O1W	0.036 (3)	0.024 (3)	0.023 (3)	0.003 (3)	0.007 (2)	−0.004 (2)
O2W	0.015 (2)	0.021 (3)	0.020 (3)	0.002 (2)	0.005 (2)	−0.003 (2)
O3W	0.025 (3)	0.031 (3)	0.018 (2)	0.003 (2)	0.009 (2)	0.001 (2)
O4W	0.021 (2)	0.022 (3)	0.012 (2)	0.005 (2)	0.0032 (19)	−0.002 (2)
O5W	0.029 (3)	0.057 (4)	0.025 (3)	0.002 (3)	0.003 (3)	0.015 (3)
O6W	0.030 (3)	0.018 (3)	0.019 (2)	−0.002 (2)	0.008 (2)	0.001 (2)
O7W	0.021 (3)	0.038 (3)	0.016 (3)	−0.003 (2)	0.007 (2)	0.002 (2)
O8W	0.020 (2)	0.024 (3)	0.013 (2)	−0.006 (2)	0.003 (2)	−0.004 (2)

### Geometric parameters (Å, º)

**Table:**

Mo1—O7B	1.980 (4)	K1—O5W^ii^	3.076 (7)
Mo1—O12B	1.904 (4)	K1—O20T^iv^	3.173 (6)
Mo2—O7B	1.985 (4)	K1—O23T	3.415 (6)
Mo2—O8B	1.879 (4)	K1—Mo4^iv^	3.797 (4)
Mo3—O8B	1.946 (4)	K2—O20T	2.828 (6)
Mo3—O9B	1.946 (4)	K2—O21T	2.858 (7)
Mo4—O9B	1.892 (4)	K2—O5W^i^	2.893 (8)
Mo4—O10B	1.987 (4)	K2—O17T^v^	3.229 (7)
Mo5—O10B	1.990 (4)	K2—O20T^vi^	3.237 (7)
Mo5—O11B	1.885 (4)	K2—Mo3^v^	3.888 (5)
Mo6—O11B	1.950 (4)	Na1—O3W^vii^	2.306 (6)
Mo6—O12B	1.927 (4)	Na1—O5W	2.329 (6)
Mo1—O14T	1.698 (4)	Na1—O4W^viii^	2.335 (5)
Mo1—O13T	1.708 (5)	Na1—O1W^ii^	2.361 (6)
Mo1—O1C	2.285 (4)	Na1—O18T^vii^	2.471 (5)
Mo1—O6C	2.314 (4)	Na1—O24T	2.483 (5)
Mo2—O16T	1.703 (4)	Na1—H5A	2.68 (7)
Mo2—O15T	1.719 (4)	O1C—H1	0.85 (3)
Mo2—O1C	2.264 (4)	O2C—H2	0.81 (3)
Mo2—O2C	2.308 (4)	O3C—H3	0.84 (3)
Mo3—O18T	1.698 (4)	O4C—H4	0.84 (3)
Mo3—O17T	1.698 (4)	O5C—H5	0.83 (3)
Mo3—O3C	2.302 (4)	O6C—H6	0.82 (3)
Mo3—O2C	2.316 (4)	O7B—O10B^ix^	2.430 (5)
Mo4—O20T	1.698 (4)	O7B—H7	1.213 (16)
Mo4—O19T	1.712 (4)	O13T—K1^ii^	2.950 (6)
Mo4—O4C	2.264 (4)	O17T—K1^ix^	2.852 (6)
Mo4—O3C	2.321 (4)	O17T—K2^x^	3.228 (7)
Mo5—O21T	1.704 (4)	O18T—Na1^iii^	2.471 (5)
Mo5—O22T	1.718 (4)	O19T—K1^xi^	3.006 (6)
Mo5—O4C	2.266 (4)	O20T—K1^xi^	3.172 (6)
Mo5—O5C	2.305 (4)	O20T—K2^vi^	3.237 (7)
Mo6—O24T	1.696 (5)	O1W—H1A	0.85 (3)
Mo6—O23T	1.708 (4)	O1W—H1B	0.86 (3)
Mo6—O6C	2.287 (4)	O2W—H2A	0.85 (3)
Mo6—O5C	2.324 (4)	O2W—H2B	0.83 (3)
Co1—O2C	1.906 (4)	O3W—H3A	0.83 (3)
Co1—O1C	1.908 (4)	O3W—H3B	0.83 (3)
Co1—O6C	1.910 (4)	O4W—H4A	0.84 (3)
Co1—O5C	1.911 (4)	O4W—H4B	0.84 (3)
Co1—O3C	1.911 (4)	O5W—H5A	0.84 (3)
Co1—O4C	1.920 (4)	O5W—H5B	0.85 (3)
K1—O17T^i^	2.852 (6)	O6W—H6A	0.83 (3)
K1—O12B	2.871 (6)	O6W—H6B	0.84 (3)
K1—O2W	2.943 (6)	O7W—H7A	0.81 (3)
K1—O13T^ii^	2.950 (6)	O7W—H7B	0.82 (3)
K1—K2^iii^	2.956 (6)	O8W—H8A	0.82 (3)
K1—O19T^iv^	3.006 (6)	O8W—H8B	0.83 (3)
			
Mo1—O7B—Mo2	118.12 (19)	O1C—Co1—O6C	84.32 (18)
Mo2—O8B—Mo3	119.0 (2)	O2C—Co1—O5C	179.2 (2)
Mo4—O9B—Mo3	119.3 (2)	O1C—Co1—O5C	95.56 (18)
Mo4—O10B—Mo5	117.23 (19)	O6C—Co1—O5C	83.83 (18)
Mo5—O11B—Mo6	118.3 (2)	O2C—Co1—O3C	83.78 (17)
Mo1—O12B—Mo6	117.4 (2)	O1C—Co1—O3C	96.02 (18)
O14T—Mo1—O13T	106.9 (2)	O6C—Co1—O3C	179.6 (2)
O14T—Mo1—O12B	98.0 (2)	O5C—Co1—O3C	96.07 (17)
O13T—Mo1—O12B	103.87 (19)	O2C—Co1—O4C	96.82 (18)
O14T—Mo1—O7B	99.3 (2)	O1C—Co1—O4C	179.5 (2)
O13T—Mo1—O7B	94.91 (19)	O6C—Co1—O4C	95.66 (18)
O12B—Mo1—O7B	149.46 (18)	O5C—Co1—O4C	83.99 (18)
O14T—Mo1—O1C	95.71 (19)	O3C—Co1—O4C	83.99 (18)
O13T—Mo1—O1C	154.61 (19)	O17T^i^—K1—O12B	111.63 (18)
O12B—Mo1—O1C	83.77 (16)	O17T^i^—K1—O2W	98.47 (17)
O7B—Mo1—O1C	69.63 (15)	O12B—K1—O2W	73.14 (15)
O14T—Mo1—O6C	161.41 (19)	O17T^i^—K1—O13T^ii^	141.7 (2)
O13T—Mo1—O6C	91.12 (19)	O12B—K1—O13T^ii^	76.40 (16)
O12B—Mo1—O6C	72.70 (16)	O2W—K1—O13T^ii^	119.31 (18)
O7B—Mo1—O6C	83.21 (16)	O17T^i^—K1—O19T^iv^	72.37 (15)
O1C—Mo1—O6C	67.73 (15)	O12B—K1—O19T^iv^	100.20 (17)
O16T—Mo2—O15T	107.1 (2)	O2W—K1—O19T^iv^	166.19 (19)
O16T—Mo2—O8B	99.4 (2)	O13T^ii^—K1—O19T^iv^	69.30 (14)
O15T—Mo2—O8B	102.4 (2)	K2^iii^—K1—O19T^iv^	110.61 (18)
O16T—Mo2—O7B	99.8 (2)	O17T^i^—K1—O5W^ii^	139.4 (2)
O15T—Mo2—O7B	93.30 (18)	O12B—K1—O5W^ii^	107.96 (17)
O8B—Mo2—O7B	150.30 (17)	O2W—K1—O5W^ii^	84.68 (18)
O16T—Mo2—O1C	93.51 (18)	O13T^ii^—K1—O5W^ii^	57.22 (15)
O15T—Mo2—O1C	155.67 (18)	K2^iii^—K1—O5W^ii^	74.83 (17)
O8B—Mo2—O1C	86.44 (16)	O19T^iv^—K1—O5W^ii^	109.03 (18)
O7B—Mo2—O1C	70.01 (15)	O17T^i^—K1—O20T^iv^	74.43 (15)
O16T—Mo2—O2C	159.74 (18)	O12B—K1—O20T^iv^	149.92 (19)
O15T—Mo2—O2C	93.03 (17)	O2W—K1—O20T^iv^	136.46 (19)
O8B—Mo2—O2C	73.14 (16)	O13T^ii^—K1—O20T^iv^	81.66 (16)
O7B—Mo2—O2C	81.06 (16)	K2^iii^—K1—O20T^iv^	63.66 (15)
O1C—Mo2—O2C	67.59 (15)	O19T^iv^—K1—O20T^iv^	52.21 (13)
O18T—Mo3—O17T	107.0 (2)	O5W^ii^—K1—O20T^iv^	75.85 (16)
O18T—Mo3—O9B	100.8 (2)	O17T^i^—K1—O23T	65.64 (14)
O17T—Mo3—O9B	97.41 (19)	O12B—K1—O23T	50.53 (12)
O18T—Mo3—O8B	97.0 (2)	O2W—K1—O23T	99.51 (16)
O17T—Mo3—O8B	100.8 (2)	O13T^ii^—K1—O23T	99.58 (16)
O9B—Mo3—O8B	149.52 (18)	K2^iii^—K1—O23T	130.99 (18)
O18T—Mo3—O3C	95.35 (18)	O19T^iv^—K1—O23T	67.49 (14)
O17T—Mo3—O3C	156.84 (19)	O5W^ii^—K1—O23T	154.08 (18)
O9B—Mo3—O3C	71.88 (15)	O20T^iv^—K1—O23T	114.93 (16)
O8B—Mo3—O3C	82.01 (16)	O20T—K2—O17T^v^	174.2 (2)
O18T—Mo3—O2C	159.84 (19)	O21T—K2—O17T^v^	107.0 (2)
O17T—Mo3—O2C	91.83 (18)	O5W^i^—K2—O17T^v^	101.0 (2)
O9B—Mo3—O2C	83.43 (16)	K1^vii^—K2—O17T^v^	54.70 (13)
O8B—Mo3—O2C	71.85 (16)	O20T—K2—O20T^vi^	115.0 (2)
O3C—Mo3—O2C	67.00 (14)	O21T—K2—O20T^vi^	172.2 (2)
O20T—Mo4—O19T	106.0 (2)	O5W^i^—K2—O20T^vi^	77.54 (19)
O20T—Mo4—O9B	99.62 (19)	K1^vii^—K2—O20T^vi^	61.43 (14)
O19T—Mo4—O9B	103.35 (19)	O17T^v^—K2—O20T^vi^	68.79 (15)
O20T—Mo4—O10B	98.97 (19)	O3W^vii^—Na1—O5W	150.1 (2)
O19T—Mo4—O10B	94.22 (18)	O3W^vii^—Na1—O4W^viii^	95.9 (2)
O9B—Mo4—O10B	149.70 (18)	O5W—Na1—O4W^viii^	106.6 (2)
O20T—Mo4—O4C	92.86 (19)	O3W^vii^—Na1—O1W^ii^	90.2 (2)
O19T—Mo4—O4C	157.33 (18)	O5W—Na1—O1W^ii^	113.2 (2)
O9B—Mo4—O4C	85.36 (17)	O4W^viii^—Na1—O1W^ii^	78.19 (19)
O10B—Mo4—O4C	69.99 (15)	O3W^vii^—Na1—O18T^vii^	80.04 (18)
O20T—Mo4—O3C	159.40 (18)	O5W—Na1—O18T^vii^	83.7 (2)
O19T—Mo4—O3C	94.43 (17)	O4W^viii^—Na1—O18T^vii^	82.32 (18)
O9B—Mo4—O3C	72.33 (16)	O1W^ii^—Na1—O18T^vii^	157.2 (2)
O10B—Mo4—O3C	81.98 (15)	O3W^vii^—Na1—O24T	80.59 (18)
O4C—Mo4—O3C	67.98 (15)	O5W—Na1—O24T	85.70 (19)
O21T—Mo5—O22T	106.6 (2)	O4W^viii^—Na1—O24T	156.9 (2)
O21T—Mo5—O11B	99.98 (19)	O1W^ii^—Na1—O24T	78.99 (19)
O22T—Mo5—O11B	103.08 (19)	O18T^vii^—Na1—O24T	119.07 (18)
O21T—Mo5—O10B	99.22 (19)	Co1—O1C—Mo2	105.09 (18)
O22T—Mo5—O10B	93.26 (18)	Co1—O1C—Mo1	104.52 (18)
O11B—Mo5—O10B	149.96 (17)	Mo2—O1C—Mo1	96.78 (15)
O21T—Mo5—O4C	94.70 (18)	Co1—O2C—Mo2	103.52 (18)
O22T—Mo5—O4C	154.94 (18)	Co1—O2C—Mo3	104.42 (17)
O11B—Mo5—O4C	85.68 (16)	Mo2—O2C—Mo3	90.92 (15)
O10B—Mo5—O4C	69.90 (15)	Co1—O3C—Mo3	104.78 (17)
O21T—Mo5—O5C	161.85 (17)	Co1—O3C—Mo4	103.09 (17)
O22T—Mo5—O5C	91.50 (17)	Mo3—O3C—Mo4	91.48 (15)
O11B—Mo5—O5C	73.56 (16)	Co1—O4C—Mo4	104.91 (18)
O10B—Mo5—O5C	81.08 (16)	Co1—O4C—Mo5	104.39 (18)
O4C—Mo5—O5C	68.21 (15)	Mo4—O4C—Mo5	97.09 (15)
O24T—Mo6—O23T	107.2 (2)	Co1—O5C—Mo5	103.25 (18)
O24T—Mo6—O12B	101.4 (2)	Co1—O5C—Mo6	103.77 (17)
O23T—Mo6—O12B	97.07 (19)	Mo5—O5C—Mo6	90.68 (15)
O24T—Mo6—O11B	97.3 (2)	Co1—O6C—Mo6	105.19 (18)
O23T—Mo6—O11B	100.39 (19)	Co1—O6C—Mo1	103.42 (18)
O12B—Mo6—O11B	149.29 (18)	Mo6—O6C—Mo1	90.73 (15)
O24T—Mo6—O6C	94.50 (19)	Mo1—O7B—O10B^ix^	119.0 (2)
O23T—Mo6—O6C	157.64 (19)	Mo2—O7B—O10B^ix^	122.7 (2)
O12B—Mo6—O6C	72.95 (16)	H1A—O1W—H1B	103 (4)
O11B—Mo6—O6C	81.53 (16)	H2A—O2W—H2B	106 (4)
O24T—Mo6—O5C	159.63 (19)	H3A—O3W—H3B	109 (4)
O23T—Mo6—O5C	92.00 (18)	H4A—O4W—H4B	108 (4)
O12B—Mo6—O5C	82.35 (16)	H5A—O5W—H5B	107 (5)
O11B—Mo6—O5C	72.04 (16)	H6A—O6W—H6B	107 (4)
O6C—Mo6—O5C	67.21 (14)	H7A—O7W—H7B	110 (5)
O2C—Co1—O1C	83.63 (18)	H8A—O8W—H8B	112 (4)
O2C—Co1—O6C	96.33 (18)		

Symmetry codes: (i) *x*−1/2, −*y*+1/2, *z*−1/2; (ii) −*x*+1, −*y*, −*z*+1; (iii) *x*+1/2, −*y*+1/2, *z*−1/2; (iv) −*x*+1/2, *y*−1/2, −*z*+1/2; (v) *x*−1, *y*, *z*; (vi) −*x*, −*y*+1, −*z*+1; (vii) *x*−1/2, −*y*+1/2, *z*+1/2; (viii) −*x*+1/2, *y*−1/2, −*z*+3/2; (ix) *x*+1/2, −*y*+1/2, *z*+1/2; (x) *x*+1, *y*, *z*; (xi) −*x*+1/2, *y*+1/2, −*z*+1/2.

### Hydrogen-bond geometry (Å, º)

**Table:**

*D*—H···*A*	*D*—H	H···*A*	*D*···*A*	*D*—H···*A*
O1*C*—H1···O2*W*	0.85 (3)	1.81 (3)	2.639 (6)	167 (7)
O2*C*—H2···O22*T*^ix^	0.81 (3)	1.98 (3)	2.787 (6)	170 (7)
O3*C*—H3···O6*W*	0.84 (3)	1.99 (4)	2.775 (6)	154 (6)
O4*C*—H4···O8*W*	0.84 (3)	1.81 (3)	2.627 (6)	165 (7)
O5*C*—H5···O15*T*^i^	0.83 (3)	1.99 (3)	2.822 (6)	171 (7)
O6*C*—H6···O7*W*	0.82 (3)	1.96 (3)	2.761 (6)	165 (7)
O7*B*—H7···O10*B*^ix^	1.21 (2)	1.22 (2)	2.430 (5)	175 (6)
O1*W*—H1*B*···O14*T*	0.86 (3)	1.89 (4)	2.731 (7)	163 (8)
O1*W*—H1*A*···O16*T*	0.85 (3)	2.18 (5)	2.878 (7)	140 (6)
O2*W*—H2*A*···O8*W*^iii^	0.85 (3)	1.91 (3)	2.757 (6)	176 (7)
O2*W*—H2*B*···O15*T*^i^	0.83 (3)	2.13 (4)	2.841 (6)	145 (6)
O3*W*—H3*B*···O19*T*	0.83 (3)	2.01 (3)	2.792 (7)	156 (6)
O3*W*—H3*A*···O1*W*^i^	0.83 (3)	2.02 (4)	2.784 (7)	154 (8)
O4*W*—H4*A*···O23*T*^ix^	0.84 (3)	1.97 (3)	2.800 (6)	167 (7)
O4*W*—H4*B*···O9*B*	0.84 (3)	1.91 (3)	2.734 (6)	167 (7)
O6*W*—H6*B*···O3*W*	0.84 (3)	1.88 (3)	2.709 (7)	169 (7)
O6*W*—H6*A*···O11*B*^iii^	0.83 (3)	2.31 (6)	2.921 (6)	131 (6)
O7*W*—H7*A*···O8*B*^vii^	0.81 (3)	2.42 (6)	2.937 (6)	122 (6)
O7*W*—H7*B*···O6*W*^vii^	0.82 (3)	2.00 (3)	2.811 (7)	166 (8)
O8*W*—H8*B*···O4*W*	0.83 (3)	1.88 (3)	2.697 (7)	168 (7)
O8*W*—H8*A*···O7*W*	0.82 (3)	2.01 (4)	2.761 (8)	151 (7)

Symmetry codes: (i) *x*−1/2, −*y*+1/2, *z*−1/2; (iii) *x*+1/2, −*y*+1/2, *z*−1/2; (vii) *x*−1/2, −*y*+1/2, *z*+1/2; (ix) *x*+1/2, −*y*+1/2, *z*+1/2.
